# A novel PID controller for BLDCM speed control using dual fuzzy logic systems with HSA optimization

**DOI:** 10.1038/s41598-022-15487-x

**Published:** 2022-07-04

**Authors:** Tingting Wang, Hongzhi Wang, Chuhang Wang, Huangshui Hu

**Affiliations:** 1 College of Mechatronic Engineering, Changchun University of Technology, Changchun, 130012 China; 2 College of Computer Science and Engineering, Changchun University of Technology, Changchun, 130012 China; 3 College of Computer Science and Technology, Changchun Normal University, Changchun, 130032 China

**Keywords:** Engineering, Mathematics and computing

## Abstract

In order to enhance the speed control performance of the brushless DC motor (BLDCM), a novel proportion integration differentiation (PID) is proposed in this paper by using dual fuzzy logic systems (FLSs) with harmony search algorithm (HSA) optimization, which is called DFPID-HSA. Firstly, the FLS1 in DFPID-HSA locks the three coefficients of the PID controller in an extensive range on the basis of the system error and error change rate. Then, the FLS2 is optimized by HSA (HSA-F2) to obtain the precise correction of the three coefficients. To get the optimal global harmony better, the improved dynamic adjustment mode is used for the pitch adjustment rate (PAR) and distance bandwidth (BW) in HSA, and the triple selection method is adopted in the composition harmony section to realize the global search. Finally, DFPID-HSA provides the optimal supply control signal to BLDCM so that it can control the speed effectively. Moreover, the stability of the system is analyzed by the pole, Lyapunov, and Nyquist determination methods. And the sensitivity analysis of DFPID-HSA is carried out under the condition of different motor’s mechanical parameters to check its robustness. In addition, the superiority of DFPID-HSA is verified by MATLAB simulation and experiment platform.

## Introduction

Brushless direct current motor (BLDCM) has been successfully applied to electric vehicles^1,2^, aerospace^3,4^, photovoltaic water pumps^5^, and other industrial and agricultural fields due to its advantages such as good speed regulation performance, high power density, high reliability, and easy control^6^. Given the broad application of BLDCM, the research on its control problem is of great importance. In the face of the progress and development of science and technology, people's demand for motor control problems also increases day by day. For decades, experts and scholars have proposed various intelligent control strategies to obtain better control performance of motors^7^.

For BLDCM control systems, PID is one of the most classic control strategies. Generally, P (proportional), I (integral), and D (differential) can be make up many forms. For example, PI, PD, PID have been successfully implemented in the BLDCM’s speed control^8,9^. Although the traditional PID structure can be easily implemented in the control system of the motor, its drawbacks, such as non-deterministic parameters and nonlinear problems, lead to the system being unable to achieve the optimal control effect. Therefore, many intelligent algorithms optimized PID controllers are put forward. Gobinath and Mu et al.^10,11^ adopt neural networks to optimize PID form controllers. Although the control performance is improved, the neural network training process is online or offline, with high computational complexity and slow response speed. Dat and Xie et al.^12,13^ use particle swarm optimization algorithm to optimize PID structure controllers, and the control performance is improved to a large extent. Still, it is difficult for the particle swarm algorithm to find the optimal solution through particle or individual iteration. Demirtas^14^ proposed the genetic algorithm to optimize the PI controller’s gains, but its initial population is challenging to determine. However, fuzzy logic control does not require a precise system model, and only calculations ground on expert knowledge bases. Therefore, optimization methods ground on fuzzy logic control has better control effects than other algorithms in most cases^15,16^. For example, He et al.^17^ proposed a new fuzzy self-tuning PID optimal controller based on the analysis of the basic working principle of brushless dc motor. The controller output switches power MOSFET devices by changing the duty ratio of PWM control signal to realizes the speed control of brushless DC motor. Yin et al.^18^ designed a fuzzy parameter adaptive PI control algorithm based on the speed loop of brushless DC motor, which has good control effect and robustness and can ensure the stable operation of the system under under variable speed conditions.

The superiority of the fuzzy logic control optimization algorithm is obvious, yet its shortcomings are also inevitable. The definition of its knowledge rule base is not scientific, so its adjustment of PID parameters still needs to be optimized. In^19^, an ANFIS controller with fuzzy PID online supervision is adopted to realize speed control of BLDCM, which has good performance under various driving conditions. However, it still fluctuates slightly in the steady-state. Premkumar and Valdez et al.^9,20^ proposed using bat algorithm, particle swarm, and other group optimization algorithms to adjust the fuzzy PID controller adaptively. In^21^, the adaptive fuzzy neural network control algorithm is adopted to realize the speed tracking of the BLDCM drive system. Rubaai et al.^22^ adopted the genetic algorithm to optimize the scale factor of the output variable of the fuzzy PID controller. In^23^, a speed control method of BLDCM based on the genetic algorithm optimizing fuzzy PID membership function and rule base is proposed. All the above algorithms have better control effects than the traditional fuzzy PID control method, and they also have the limitations of the algorithm mentioned in the previous section. Harmony search algorithm (HSA) is a newly published heuristic global search algorithm, which has been successfully implemented in many combinatorial optimization solution problems^24,25^, such as solving continuous optimization problems^26^, solving unconstrained problems^27^, and also in field of motor^28,29^. It is shown that the harmony search algorithm has better performance than the genetic algorithm, simulated annealing algorithm, and tabu search algorithm, etc. In^30^, an optimization method combining harmony search algorithm with fuzzy logic is successfully proposed, and the superiority of the process is verified.

Based on the descriptions of the above algorithm mentioned, this paper proposes a novel PID controller using dual FLSs with HSA optimization called DFPID-HSA to enhance the various speed control performance of BLDCM. The major contributions of this paper are as follows.

1. DFPID-HSA adopts dual FLSs, in which the FLS1 locks the three coefficients of the PID controller in an extensive range on the basis of the system error and the error change rate. Then, the FLS2 is optimized by HSA (HSA-F2) to obtain the precise correction of the three coefficients.
2. To better obtain the optimal global harmony, the PAR and BW of HSA adopt the improved dynamic adjustment mode. In the composition harmony section, the triple selection method is used to achieve the optimal global search. Finally, DFPID-HSA provides the optimal control signal to BLDCM to realize the speed control of BLDCM.
3. The stability of the proposed controller is analyzed by the pole determination method, the Lyapunov determination method, and the Nyquist determination method. Then the system has been demonstrated to be closed-loop stable.
4. The performance indicators about steady-state, transient, and integral of DFPID-HSA are compared with the deep perceptron neural network optimized fuzzy PID controller(DPNN-FuzzyPID)^10^, the fuzzy logic PID controller optimized by genetic algorithm (GA-PID-FLC)^23^, the fuzzy logic PID controller based on particle swarm optimization (PSO-FuzzyPID)^31^, PID controller with fuzzy logic regulation (FuzzyPID)^15^, and conventional PID controller (PID) by Matlab. The superiority of DFPID-HSA in BLDCM speed control is verified. And the sensitivity analyses of DFPID-HSA are carried out under mechanical parameters variations of the motor to check its robustness.
5. The BLDCM drive system experimental platform is built. Under three experimental conditions, it is verified that DFPID-HSA still maintains its superiority and can achieve excellent control of the BLDCM, which proves the feasibility of the algorithm.

Other organizational structures for this article are as follows: the second section presents the establishment of the BLDCM mathematical model. The third section describes the principle of the proposed DFPID-HSA algorithm. In the fourth section, the simulation model of the BLDCM control system is built, and the performance comparison test of the presented algorithm is implemented. In the fifth section, the experimental platform of the BLDCM control system is built to verify the feasibility of the presented algorithm. The sixth section summarizes the article.

## Mathematical mode of BLDCM

**Figure 1:**
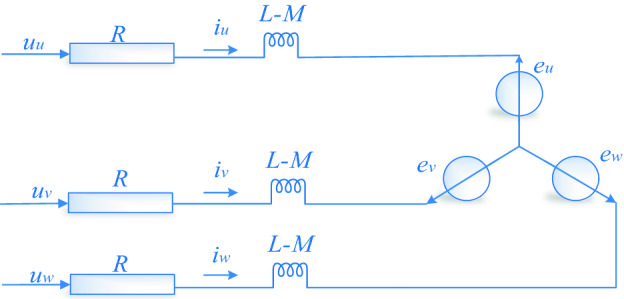
Equivalent circuit of BLDCM.

The three-phase star-connected BLDCM can be converted to the circuit diagram shown in Fig. 1. The mathematical model of an ideal motor requires the assumption that the motor body satisfies the following conditions^32^: (1) ignore the saturation of the motor iron core, (2) ignore the eddy current and hysteresis losses in the motor; (3) the current in the motor is the three-phase symmetric sinusoidal current; and (4) the effects of temperature, frequency variation, and winding damping on resistance are not considered. The three-phase winding voltage equation may be expressed as:

1 $$u_{x} = Ri_{x} + (L - M)\frac{{di_{x} }}{dt} + e_{x}$$

where, $u_{x}$, $i_{x}$, $e_{x}$ $(x = u,v,w)$ and *R* denotes the phase voltage, phase current, back electromotive force, and phase impedance of the stator windings, respectively; *L* and *M* represent the self-inductance and pairwise mutual inductance of the three-phase windings, respectively.

The electromagnetic torque generated by the stator winding is

2 $$T_{e} = (e_{u} i_{u} + e_{v} i_{v} + e_{w} i_{w} )/\omega_{m}$$

where, $\omega_{m}$ and $T_{e}$ represent the mechanical angular speed and electromagnetic torque of the BLDCM, respectively.

**Figure 2:**
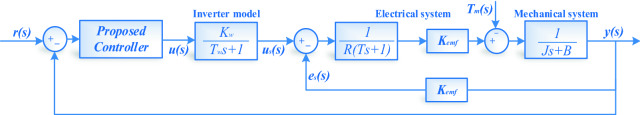
Block diagram of the speed control system for BLDCM.

The equation of motion of the BLDCM is as follows:

3 $$T_{{\text{e}}} = T_{{\text{m}}} + B\frac{{d\omega_{{\text{m}}} }}{dt} + J\omega_{{\text{m}}}$$

where, $T_{m}$, $B$ and $J$ represent load torque, damping coefficient, and moment of inertia, respectively. Hence, the characteristic equation of BLDCM can be expressed as^10,33^:

4 $$\left\{ \begin{gathered} u_{s} (t) = Ri(t) + L\frac{di(t)}{{dt}} + e_{s} (t) \hfill \\ e_{s} (t) = K_{emf} \omega_{m} (t) \hfill \\ T_{e} (t) = K_{emf} i(t) \hfill \\ T_{e} (t) = J\frac{{d\omega_{m} (t)}}{dt} + B\omega_{m} (t) + T_{m} \hfill \\ \end{gathered} \right.$$

where, *K*_*emf*_ is the back electromotive force constant. Figure 2 is the block diagram of the speed control system for BLDCM. The proposed controller mainly realizes tracking control for the speed of the BLDCM. Table 1 gives the basic parameters of BLDCM and inverter. From the characteristic equation of the BLDCM given in Eq. (4), the transfer function model of the BLDCM is deducted as,

5 $$\frac{y(s)}{{u_{s} (s)}} = \frac{{K_{emf} }}{{R(Ts + 1)(Js + B) + K_{emf}^{2} }}$$

**Table 1:** Basic parameters of BLDCM.

Parameters	Value	Units
Stator phase resistance *R*	2.875	Ω
Stator phase inductance *L*	0.0085	H
Flux linkage established by magnets *λ*	0.175	V-s
Voltage constant	0.1466	V/(r/min)
Torque constant	1.4	N (m/A)
Moment of inertia *J*	0.0008	(kg m^2^)
Friction factor *B*	0.001	N (m s/rad)
Pole pairs *P*	4	–
Inverter gain *K*_*w*_	500	–
Inverter time constant *T*_*w*_	5 × 10^−6^	s

The transfer function model of the PWM inverter is given as,

6 $$\frac{u(s)}{{u_{s} (s)}} = \frac{{K_{w} }}{{T_{w} s + 1}}$$

## Proposed DFPID-HSA controller

Aiming at the speed control problem for BLDCM, this paper proposes an HSA optimized dual fuzzy logic systems-based PID controller called DFPID-HSA. The specific control system construction is given in Fig. 2. Firstly, the FLS1 in DFPID-HSA locks the proportional coefficient *K*_*P*1_, integral coefficient *K*_*I*1_, and differential coefficient *K*_*D*1_ of PID controller in a wide range on the basis of the system error *e* and error change rate *ec*. Then, the accurate correction value *k*_*p'*_*/k*_*i'*_*/k*_*d'*_ of *K*_*P*1_*/K*_*I*1_*/K*_*D*1_ is obtained by HSA optimized FLS2. In order to get the optimal global harmony better, the PAR and BW in HSA adopt the improved dynamic adjustment mode, and the triple selection method is adopted in the composition harmony section to realize the optimal global search. Finally, DFPID-HSA provides the optimal control signal *u(t)* to BLDCM to realize the speed control.

**Figure 3:**
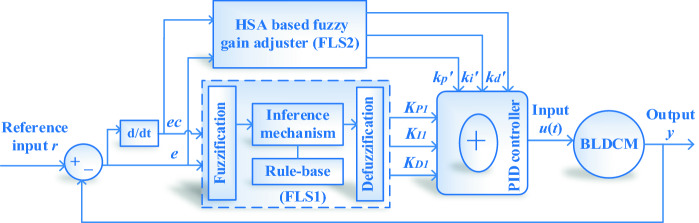
The architecture of the BLDCM control system.

According to the structure in Fig. 3, it is known that $e = y - r$, $ec = {{de} \mathord{\left/ {\vphantom {{de} {dt}}} \right. \kern-\nulldelimiterspace} {dt}}$, and the control signal *u*(*t*) can be given as

7 $$u(t) = K_{P} e + K_{I} \int {edt} + K_{D} {\raise0.7ex\hbox{${de}$} \!\mathord{\left/ {\vphantom {{de} {dt}}}\right.\kern-\nulldelimiterspace} \!\lower0.7ex\hbox{${dt}$}}$$

where, *K*_*P*_, *K*_*I*_, and *K*_*D*_ in A are determined by the output parameters *K*_*P*1_*/K*_*I*1_*/K*_*D*1_ of FLS1 in DFPID-HSA, and the output parameters *k*_*p'*_*/k*_*i'*_*/k*_*d'*_ of HSA Optimized FLS2.

8 $$\left\{ \begin{gathered} K_{P} = K_{P1} + k_{{p^{\prime}}} \hfill \\ K_{I} = K_{I1} + k_{{i^{\prime}}} \hfill \\ K_{D} = K_{D1} + k_{{d^{\prime}}} \hfill \\ \end{gathered} \right.$$

### Fuzzy logic system

The fundamental structure of the fuzzy logic system is given in the dashed box in Fig. 2, and it mainly consists of the below four parts^34^:

- Fuzzification
  The role of fuzzification is to transform the precise input quantity into fuzzification quantity. The input contains external reference input, system output or state, etc.
- Knowledge bases
  The knowledge bases include the knowledge in the specific application field and the required control objectives. It mainly consists of two parts: databases and fuzzy control rule bases.
- Fuzzy Inference engine
  The fuzzy inference engine is the kernel of FLS, which has the inference capacity of simulating humans ground on fuzzy concepts. The inference process is ground on the implication relationship and inference rules in fuzzy logic.
- Clarification
  The role of clarification is to convert the fuzzy quantity (control quantity) got by fuzzy inference engine into the precise quantity of practical application control.

In DFPID-HSA, both FLS1 and FLS2 adopt dual-input dual-output Mamdani controllers. The fuzzification process of FLS1 and FLS2 is mainly to convert the actual values of the system speed error *e* and error change rate *ec* into the corresponding fuzzy values according to the fuzzy domains and membership functions. The fuzzy domain of input and output variables in FLS1 is: $e,ec = [ - 3,3]$, $K_{P1} ,K_{I1} ,K_{D1} = [0,60]$; the fuzzy domain of input and output variables in FLS2 is: $e,ec = [ - 1,1]$, $k_{{p^{\prime}}} ,k_{{i^{\prime}}} ,k_{{d^{\prime}}} = [0,6]$. The fuzzy language set of FLS1 and FLS2 input variables is {NB, NM, NS, ZO, PS, PM, PB} = {“negative big”, “negative middle”, “negative small”, “zero”, “positive small”, “positive middle”, “positive big”}; The fuzzy language set of FLS1 and FLS2 output variables is {VS, MS, S, M, B, MB, VB} = {“very small”, “medium small”, “small”, “medium”, “big”, “medium big”, “very big”}^35^.

**Figure 4:**
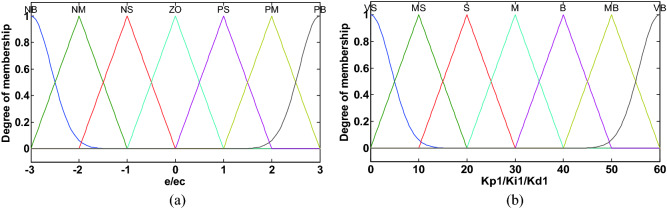
Membership function of FLS1: (**a**) input variables (**b**) output variables.

**Figure 5:**
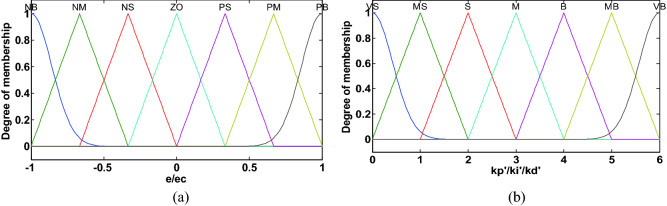
Membership function of FLS2: (**a**) input variables (**b**) output variables.

The membership functions of input and output variables of FLS1 and FLS2 are shown in Figs. 4 and 5, respectively. In this paper, the membership functions mainly choose isosceles triangle type and Gaussian function type. The isosceles triangle has the advantages of being convenient for representation, simple for calculation, and fast for the response. The edge values of the fuzzy sets mainly adopt the Gaussian function, which makes its value smoother and more adaptive. The fuzzy rules of different output variables of FLS1 and FLS2 are shown in Table 2. The establishment of fuzzy rules refers to the experience of experts and is modified through multiple simulations^36^. Specific fuzzy rules can be written in the following form:

**Table 2:** Fuzzy rules for different output variables of FLS1 / FLS2.

*ec*	*e*						
	NB	NM	NS	ZO	PS	PM	PB
**Fuzzy rules for K**_**D1**_**/k**_**p'**_							
NB	VB	VB	MB	MB	B	B	M
NM	VB	VB	MB	B	B	M	S
NS	MB	MB	MB	B	M	S	S
ZO	MB	MB	B	M	S	MS	MS
PS	B	B	M	S	S	MS	MS
PM	B	M	S	S	MS	MS	VS
PB	M	M	MS	MS	MS	VS	VS
**Fuzzy rules for K**_**I1**_**/k**_**i'**_							
NB	VS	VS	MS	MS	S	S	M
NM	VS	VS	MS	S	S	M	S
NS	VS	MS	MS	S	M	B	B
ZO	MS	MS	S	M	B	MB	B
PS	MS	S	M	B	B	MB	MB
PM	M	M	B	B	MB	MB	VB
PB	M	M	B	MB	MB	VB	VB
**Fuzzy rules for K**_**D1**_**/k**_**d'**_							
NB	B	S	MB	VS	VS	MS	B
NM	B	S	VS	MS	MS	S	M
NS	M	S	MS	MS	S	S	M
ZO	M	S	S	S	S	S	M
PS	M	M	M	M	M	M	M
PM	MB	S	B	B	B	B	VB
PB	VB	MB	MB	MB	B	B	VB

If $e = e_{f}$ and $ec = ec_{f}$, then $K_{P1} = K_{P1f}$ and $K_{I1} = K_{I1f}$ and $K_{D1} = K_{D1f}$;

If $e \, = \, e_{f}$ and $ec = ec_{f}$, then $K_{{p^{\prime}1}} = K_{{p^{\prime}f}}$ and $K_{{i^{\prime}1}} = K_{{i^{\prime}f}}$ and $K_{{d^{\prime}1}} = K_{{d^{\prime}f}}$;

(i = 1, 2, 49; each variable representing 49 rules);

where, $e_{f}$, $ec_{f}$, $K_{P1f}$, $K_{I1f}$, $K_{D1f}$, $K_{{p^{\prime}f}}$, $K_{{i^{\prime}f}}$, $K_{{d^{\prime}f}}$ represent the fuzzy language sets of $e$, $ec$, $K_{P1}$, $K_{I1}$, $K_{D1}$, $K_{{p^{\prime}}}$, $K_{{i^{\prime}}}$, $K_{{d^{\prime}}}$.

Taking $K_{P1}$ as an example, the membership degree of the first fuzzy rule of $K_{P1}$ is

9 $$\mu _{{K_{{P11}} }} = \mu _{{NB}} (e)*\mu _{{NB}} (ec)$$

where, "$*$" means to take the smaller, i.e.

10 $$\mu _{{K_{{P11}} }} = \min \{ \mu _{{NB}} (e),\mu _{{NB}} (ec)\}$$

By analogy, the membership degrees of all fuzzy rules corresponding to $K_{P1}$ under different $e$ and $ec$ can be obtained. According to the membership degree of each fuzzy rule, the fuzzy value of $K_{P1}$ can be obtained by Clarificating with the center of gravity method

11 $$K_{{P1}} = \frac{{\sum\nolimits_{{f = 1}}^{{49}} {\mu _{{K_{{P1f}} }} (K_{{P1}} )K_{{P1f}} } }}{{\sum\nolimits_{{f = 1}}^{{49}} {\mu _{{K_{{P1f}} }} (K_{{P1}} )} }}$$

where, $K_{P1f}$ is a real value on the domain $K_{P1} = [0,60]$, $\mu _{{K_{{P1f}} }}$ Is the membership degree of corresponding fuzzy rules. Similarly, the fuzzy output value of $K_{I1}$, $K_{D1}$, $K_{{p^{\prime}}}$, $K_{{i^{\prime}}}$, $K_{{d^{\prime}}}$ in each sampling period can be obtained.

### Harmony search algorithm

**Figure 6:**
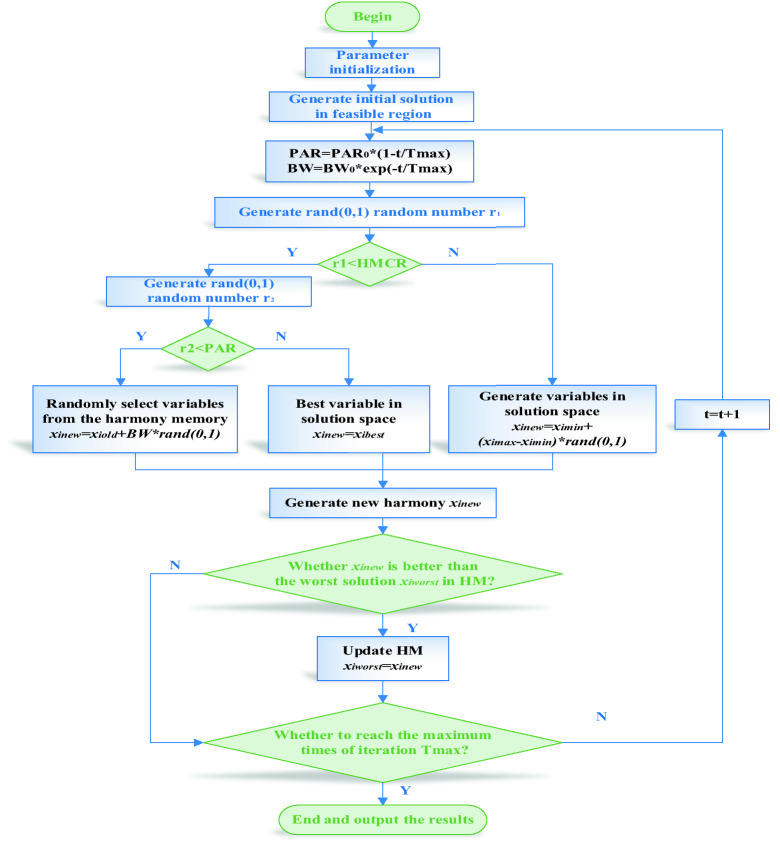
The flow chart of HSA-F2.

Harmony Search Algorithm (HSA) is a heuristic algorithm put forward by Geem et al.^37^, with strong global convergence. HSA is a simulation of the process by which musicians iteratively adjust the tones of various musical instruments to achieve the most beautiful harmony finally^38,39^. The evolution speed of HSA is faster than that of intelligent algorithms such as the genetic algorithm and has fewer mathematical requirements. HSA mainly consists of five steps^40,41^ which are as follows:

1. Define problem and parameter values
  1. This paper belongs to the problem of minimization, that is:
    12 $$\min f(X),X = \{ x_{1} ,x_{2} , \cdots ,x_{n} \} \in R^{n}$$
    where, *x*_*i*_ ∈ *X*_*i*_, *i* = 1, 2, …, *n*, *x*_*i*_ ∈ [*X*_*i* min_, *X*_*i* max_]
  2. Determine parameter values.
    Harmony memory size (HMS): Size of the harmonic population.
    Harmony memory considering rate (HMCR): Probability of taking a harmony voice from the existing population.
    Pitch adjusting rate (PAR): Probability of adjusting the harmony voice.
    Bandwidth (BW): Amplitude of pitch adjusting.
    Times of creation (Tmax): Times of adjustment (iteration).
  Obviously, a set of suitable parameters can enhance the algorithm's ability to search for the global optimal or close to the optimal region and has a high convergence speed. Where the parameter BW is the distance bandwidth of continuous design variables. An enormous BW value is conducive to search the algorithm in an extensive range, and a small BW value is suitable for adjusting the optimal solution. To better obtain the objective optimization results, the BW value in this paper decreases dynamically with the increase of iteration times. The improved dynamic adjustment method is as follows:
  13 $$BW = BW_{0} \times e^{{ - {\raise0.7ex\hbox{$t$} \!\mathord{\left/ {\vphantom {t {T\max }}}\right.\kern-\nulldelimiterspace} \!\lower0.7ex\hbox{${T\max }$}}}}$$
  where, $BW_{0}$ is the initial coefficient of pitch adjusting bandwidth, and t is the current times of iteration.
  PAR is the adjustment rate of the pitch. An enormous PAR value is conducive to transmitting the information of x_i_ to the next generation, which enhances the local development capabilities of the algorithm near x_i_. In contrast, a small PAR value capacitates the new harmony vector to expand the search range and increase the multiplicity of the harmony memory by disturbing the values of the corresponding dimensions in the harmony memory. As the times of iteration increase, it is closer to obtaining better harmony, so the probability of adjusting harmony should also be reduced. In this paper, an improved dynamic adjustment is adopted for PAR, as follows:
  14 $$PAR = PAR_{0} \times \left( {1 - {\raise0.7ex\hbox{$t$} \!\mathord{\left/ {\vphantom {t {T\max }}}\right.\kern-\nulldelimiterspace} \!\lower0.7ex\hbox{${T\max }$}}} \right)$$
  where, $PAR_{0}$ and *t* stand for the initial coefficient of pitch adjusting rate and the current times of iteration, respectively.
2. Initialization of harmony memory
  HMS harmonies $X^{1} ,X^{2} , \cdots ,X^{HMS}$ are randomly created from the solution space of X and put into the harmony memory. The matrix form of the harmony memory is:
  15 $$HM = \left[ \begin{gathered} X^{1} \hfill \\ X^{2} \hfill \\ \cdots \hfill \\ X^{HMS} \hfill \\ \end{gathered} \right]$$
  *HM* adopts external random values to prevent falling into local optimization or local convergence, as in Eq. (16)
  16 $$x_{i} = x_{i\min } + (x_{i\max } - x_{i\min } ) \times {r_{0}}$$
  where, $r_{0}$ is a random number between [0, 1].
3. Generate a new harmony
  Generate a random number $r_{1}$ between [0, 1], compare with HMCR,
  If $r_{1}$ < HMCR, take a random harmony variable from the harmony memory,
  Otherwise, a random harmonic variable is created from the solution space;
  A harmony variable is got from the above. If the harmony variable is got from the harmony memory, it is necessary to adjust it to generate a random number $r_{2}$ between [0, 1].
  If $r_{2}$ < PAR, adjust the resulting harmony variable on the basis of BW and get a new harmony variable,
  Otherwise, to avoid that the performance of the randomly generated harmony in the solution space is worse than that of the best harmony $x_{ibest}$ in *HM*, $x_{ibest}$ is used to replace the randomly generated harmony.
  Finally, we get a new harmony $x_{inew}$:
  17 $$x_{inew} = \left\{ {\begin{array}{*{20}l} {x_{iold} \pm BW \times r_{3} ,} \hfill & {r_{1} < HMCR \cup r_{2} < PAR} \hfill \\ {x_{ibest} ,} \hfill & {r_{1} < HMCR \cup r_{2} \ge PAR} \hfill \\ {x_{i\min } + \left( {x_{i\max } - x_{i\min } } \right) \times r_{0} ,} \hfill & {r_{1} \ge HMCR} \hfill \\ \end{array} } \right.$$
  where, $r_{0}$, $r_{1}$, $r_{2}$ and $r_{3}$ are random numbers between [0, 1].
4. Update harmony memory
  Evaluate $Xnew$, i.e. $f\left( {Xnew} \right)$. If it is better than the one with the worst function value in *HM*, i.e. $f\left( {Xnew} \right) < f\left( {Xworst} \right)$, then $Xnew$ will replace $Xworst$; Otherwise, no modification is made.
5. Determine the stop condition
  Repeat steps (3) and (4) until the times of creation (iteration) reach Tmax.
  In this paper, HSA is used to optimize FLS2 to obtain the accurate correction value *kp'/ki'/kd'* of FLS1 parameters. Since the BLDCM speed control system belongs to the problem of minimizing the error *e*, the cost function is defined as the Integral absolute error (IAE).
  18 $$IAE = \int_{0}^{t} {\left| {e(t)} \right|} dt$$
  The constraints of optimization variables are as follows:
  19 $$\left\{ \begin{gathered} 0 \le k_{{p^{\prime}}} \le 6 \hfill \\ 0 \le k_{{i^{\prime}}} \le 6 \hfill \\ 0 \le k_{{d^{\prime}}} \le 6 \hfill \\ \end{gathered} \right.$$
  Then, the harmony memory is
  20 $$HM = \left[ {\begin{array}{*{20}c} {k_{{p^{\prime}}}^{1} } & {k_{{i^{\prime}}}^{1} } & {k_{{d^{\prime}}}^{1} } \\ {k_{{p^{\prime}}}^{2} } & {k_{{i^{\prime}}}^{2} } & {k_{{d^{\prime}}}^{2} } \\ \cdots & \cdots & \cdots \\ {k_{{p^{\prime}}}^{HMS} } & {k_{{i^{\prime}}}^{HMS} } & {k_{{i^{\prime\prime}}}^{HMS} } \\ \end{array} } \right]$$
  The flow chart of the HSA-F2 algorithm is shown in Fig. 6, and the specific steps are shown in Table 3.

**Table 3:**
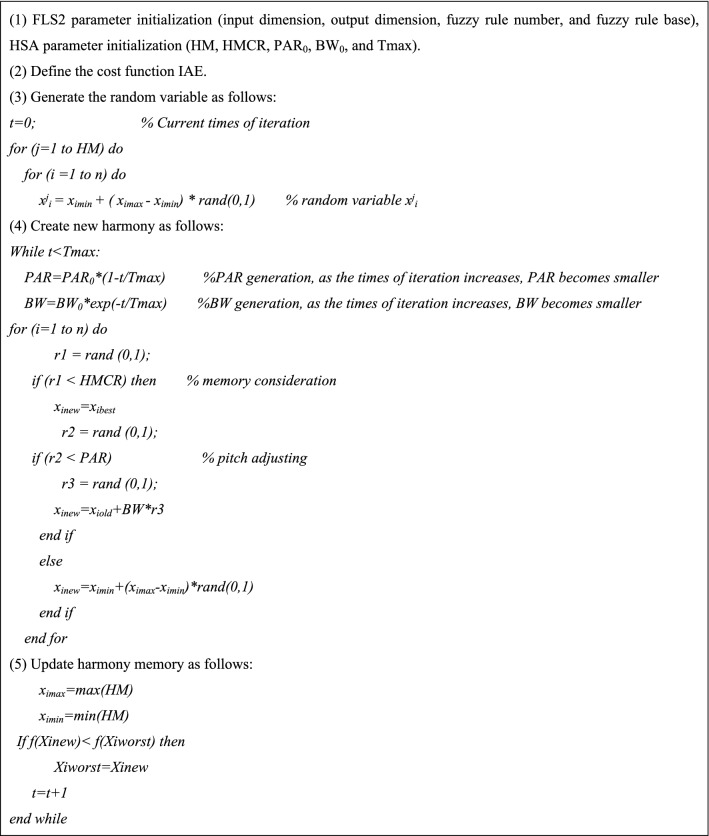
Specific steps of HSA-F2 algorithm.

## Stability analysis

This section analyzes the stability of the closed-loop system of the speed control for BLDCM based on the novel PID controller using dual fuzzy logic systems with HSA optimization. The pole determination method, Lyapunov determination method, and Nyquist determination method are used to verify the stability of the system. To test the stability, the transfer function of the closed-loop system needs to be used. Adopting bilinear transformation, the closed-loop transfer function of the optimized DFPID-HSA controlled BLDCM is provided in Eq. (21), where the transfer function of the proposed controller can be equivalent to $G_{C} (s) = {{\left( {K_{P} s + K_{I} + K_{D} s^{2} } \right)} \mathord{\left/ {\vphantom {{\left( {K_{P} s + K_{I} + K_{D} s^{2} } \right)} s}} \right. \kern-\nulldelimiterspace} s}$ according to Eqs. (7) and (8), and the load torque of the motor is taken to be zero.

21 $$\frac{y(s)}{{r(s)}} = \frac{{5.864s^{2} + 5255.61s + 397.286}}{{2.3 \times 10^{ - 9} s^{4} + 4.6 \times 10^{ - 4} s^{3} + 5.867s^{2} + 5255.635s + 397.286}}$$

### Pole determination method

**Figure 7:**
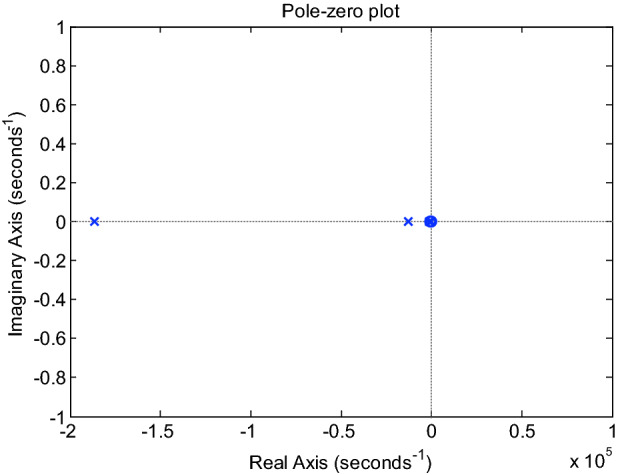
Pole-zero plot of the speed control system for the BLDCM based on DFPID-HSA.

According to the analysis of the unit step response of the higher-order system, it is the dynamic component that affects the change of system output with time. Whether the dynamic component attenuates only depends on the sign of the closed-loop pole of the system. A necessary and sufficient condition for system stability: all poles of the closed-loop system are negative real numbers or conjugate complex numbers with negative real parts. In other words, all closed-loop nodes must distribute on the left half of the imaginary axis of the S-plane^34^. Figure 7 lays out the pole-zero plot of the speed control system for the BLDCM based on DFPID-HSA. It is observed from the pole-zero plot that all the poles are on the left half of the S-plane, thus indicating that the system is stable.

On the basis of the Matlab command, the zero points (z), pole points (p) and gain (k) of the system are:

22 $$\begin{gathered} z = [\begin{array}{*{20}l} { - 896.1744} & { - 0.0756} \\ \end{array} ] \hfill \\ p = [\begin{array}{*{20}l} { - 1.8638 \times 10^{5} } & { - 0.1256 \times 10^{5} } & { - 0.0097 \times 10^{5} } & { - 0.0000} \\ \end{array} ] \hfill \\ k = [2.5496 \times 10^{9} ] \hfill \\ \end{gathered}$$

### Lyapunov determination method

Lyapunov is a Russian mathematician who derived the famous stability criteria for linear and nonlinear systems. Lyapunov theorem points out that if there is a unique $P = P^{T} > 0$ satisfying Eq. (23) for any $Q = Q^{T} > 0$, then that system is asymptotically stable^42^.

23 $$A^{T} P + PA = - Q,Q = Q^{T} ,Q = I({\text{Identity matrix}})$$

where, *Q* stands for a any positive definite matrix.

To solve the discrete-time Lyapunov equation, the state-space model matrix of the system is required. According to Eq. (21), use the tf2ss() function to obtain the state-space model matrix *A*, *B*, *C*, *D* of the speed control system for BLDCM based on DFPID-HSA,

24 $$\begin{gathered} A = \left[ {\begin{array}{*{20}c} { - 0.0000} & { - 0.0026 \times 10^{12} } & { - 2.2851 \times 10^{12} } & { - 0.1727 \times 10^{12} } \\ {0.0000} & 0 & 0 & 0 \\ 0 & {0.0000} & 0 & 0 \\ 0 & 0 & {0.0000} & 0 \\ \end{array} } \right] \hfill \\ B = \left[ {\begin{array}{*{20}c} 1 & 0 & 0 & 0 \\ \end{array} } \right]^{T} \hfill \\ C = \left[ {\begin{array}{*{20}c} 0 & {0.0025 \times 10^{12} } & {2.2850 \times 10^{12} } & {0.1727 \times 10^{12} } \\ \end{array} } \right] \hfill \\ D = \left[ 0 \right] \hfill \\ \end{gathered}$$

Utilize Eq. (23) to obtain the *P* matrix and its eigenvalues *λ*, and determine whether *P* is positive definite according to *λ*.

25 $$\lambda = \left[ {\begin{array}{*{20}c} {5.1698 \times 10^{9} } \\ {2.3395 \times 10^{12} } \\ {8.8436 \times 10^{10} } \\ {3.8910 \times 10^{7} } \\ \end{array} } \right]$$

Both *λ* are positive, which proves that *P* is positive definite, and Lyapunov criterion confirms that the speed control system of BLDCM based on DFPID-HSA is asymptotically stable.

### Nyquist determination method

Suppose the open-loop transfer function of the system be $G_{C} (s)G(s)$. If the system is open-loop stable, the necessary and sufficient condition for the stability of the closed-loop system is that when $\omega$ by $0 \to \infty$, the open-loop Nyquist curve $G_{C} (j\omega )G(j\omega )$ of the system does not enclose point $\left( { - 1,j0} \right)$, then the closed-loop system is stable. Otherwise, it is unstable^34^.

**Figure 8:**
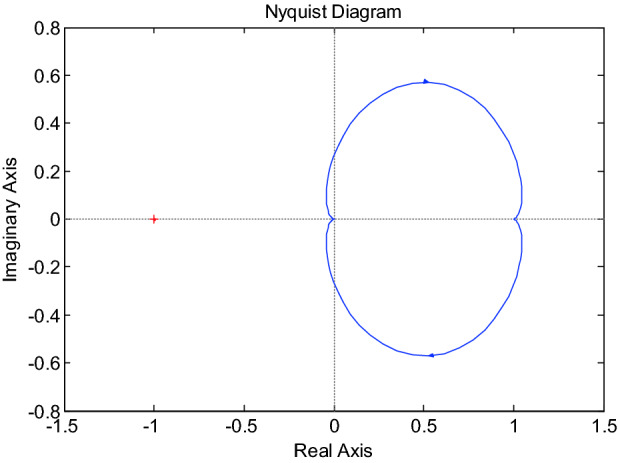
Nyquist diagram of the speed control system for the BLDCM based on DFPID-HSA.

The Nyquist diagram of the speed control system for BLDCM based on DFPID-HSA is obtained according to the nyquist() function in Matlab, see Fig. 8. It can be seen from the diagram that the system does not contain $( - 1,j0)$ point. Therefore, this paper proposes that the DFPID-HSA-based BLDCM speed control system is closed-loop stable.

## Simulation analysis

In order to verify the superiority of DFPID-HSA in BLDCM speed control, its performances are compared and analyzed with DPNN-FuzzyPID, GA-PID-FLC, PSO-FuzzyPID, FuzzyPID, and PID by MATLAB. The selection of relevant parameters in the comparison algorithms referred to the original literature, followed the selection rules of relevant data, and made reasonable adjustments in the test to ensure the fairness of comparison. The comparison performance indicators mainly include steady-state performance indicators: error (r/min, %), transient performance indicators: delay time, adjustment time, maximum overshoot/undershoot, oscillation, etc.^43^, integral performance indicators: Integral absolute error (IAE) criterion, Integral square error (ISE) criterion, Integrated time absolute error (ITAE) criterion, Integral time square error (ITSE) criterion^44,45^.

**Figure 9:**
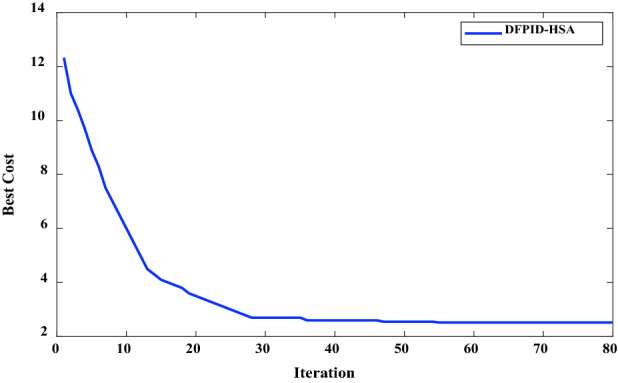
Solution convergence for DFPID-HAS.

**Figure 10:**
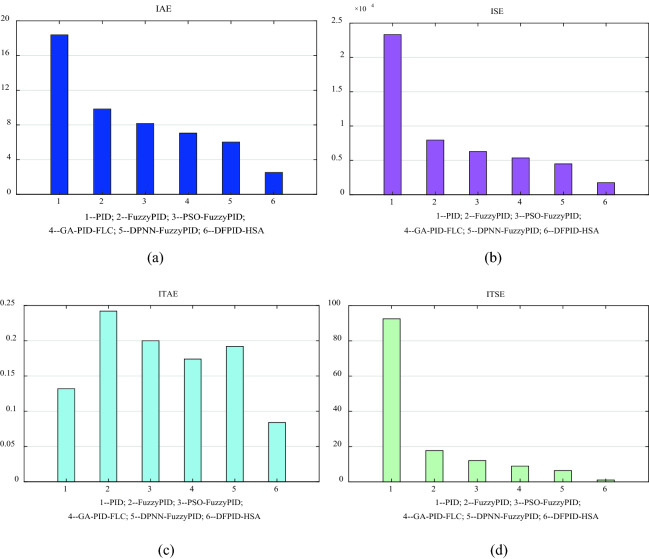
Error signal performance indicator analyses: (**a**) IAE (**b**) ISE (**c**) ITAE (**d**) ITSE.

The initialization of DFPID-HSA parameters is shown in Table 4^26^, the selection of relevant parameters mainly refers to the experience of experts, and is modified and determined through many simulations. The convergence diagram of DFPID-HSA obtained by running the system based on the corresponding parameters is shown in Fig. 9. It can be seen that the optimal cost of DFPID-HSA is obtained when the iteration reaches 55 times. The final optimized parameters Kp/Ki/Kd of the four comparison algorithms are shown in Table 5. The integral performance indicators of the four algorithms are shown in Table 6, and the error signal performance indicator analyses are shown in Fig. 10. From the comparison of the error signal performance indicators, it can be seen that DFPID-HSA is the best.

**Table 4:** Initialization parameters of DFPID-HAS.

Elements	Value
Search parameter number	3
HMS	200
HMCR	0.98
PAR_0_	0.9
BW_0_	0.01
Tmax	80

**Table 5:** The final optimized parameters of *K*_*P*_*/K*_*I*_*/K*_*D*_*.*

Algorithms/parameters	*K*_*P*_	*K*_*I*_	*K*_*D*_
PID	16.6	0.013	0.1 × 10^–5^
FuzzyPID	25.1	3.00	0.10
PSO-FuzzyPID	29.4	2.92	0.09
GA-PID-FLC	34.5	3.01	0.10
DPNN-FuzzyPID	40.2	3.21	0.09
DFPID-HSA	71.7	5.42	0.08

**Table 6:** Observed performance indices for error.

Algorithms/performance indicators	IAE	ISE	ITAE	ITSE
PID	18.38	2.3327 × 10^4^	0.132	92.52
FuzzyPID	9.84	0.7962 × 10^4^	0.242	17.73
PSO-FuzzyPID	8.17	0.6285 × 10^4^	0.200	12.07
GA-PID-FLC	7.06	0.5357 × 10^4^	0.174	8.91
DPNN-FuzzyPID	6.02	0.4493 × 10^4^	0.192	6.39
DFPID-HSA	2.52	0.1740 × 10^4^	0.084	1.02

IAE = ∫ |e(t)| dt, ISE = ∫ e^2^(t) dt, ITAE = ∫t |e(t)| dt, ITSE = ∫ te^2^(t) dt.

Considering that uncertainties such as load changes and speed changes are prone to occur in the operation of the BLDCM system, the performance comparison and analysis of the four algorithms are carried out under the following three working conditions.

### No-load condition

**Figure 11:**
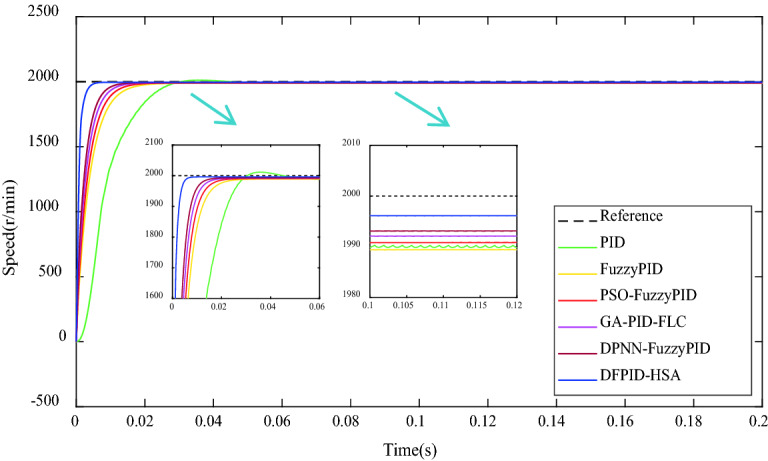
The comparison of speed response under the no-load condition.

Under the no-load condition, the target speed of BLDCM is given to be 2000 r/min. The control system is operated according to different algorithms and obtain the comparison of speed response curves, as shown in Fig. 11. As can be seen from Fig. 11 that all the five algorithms can make the system reach the ideal speed, among which PID has an evident overshoot phenomenon. In contrast, FuzzyPID, PSO-FuzzyPID, GA-PID-FLC, DPNN-FuzzyPID, and DFPID-HSA have no evident overshoot phenomenon. The maximum overshoot MP% and oscillation times N of the five algorithms meet the engineering requirements (Mp% ≤ 50%, n ≤ 1.5). Still, DFPID-HSA has the shortest delay time and settling time, and the smallest steady-state error, which shows that the control performance of DFPID-HSA is better. See Table 7 for the comparison of specific performance indicators.

**Table 7:** The comparison of performance indicators under the no-load condition.

Controllers	Transient performance indicators			Steady-state performance indicators	
	Maximum overshoot (Mp%)	Delay time (s) × 10^−3^	Settling time (s)	Steady-state error (r/min)	Steady-state error (%)
PID	1.08	12.10	0.085	9.90	0.495
FuzzyPID	–	2.65	0.043	10.64	0.532
PSO-FuzzyPID	–	2.22	0.039	9.16	0.458
GA-PID-FLC	–	1.83	0.030	7.94	0.397
DPNN-FuzzyPID	–	1.60	0.027	6.88	0.344
DFPID-HSA	–	0.60	0.015	4.10	0.205

Mp% = [Speed (max) − Speed (∞)]/Speed (∞) × 100%.

### With-load condition

**Figure 12:**
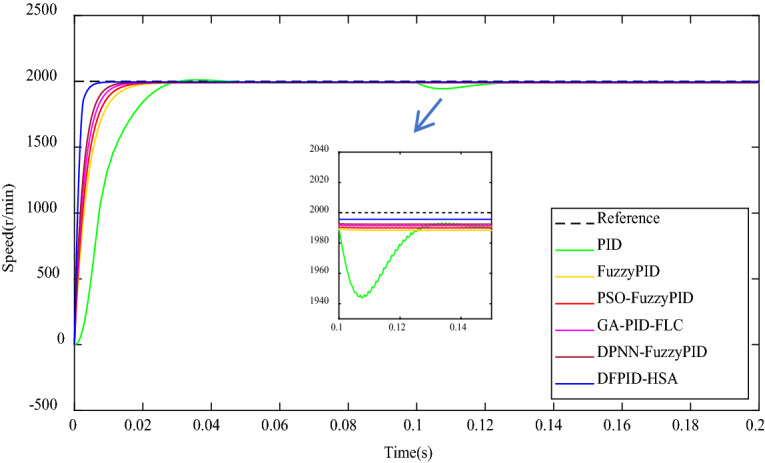
The comparison of speed response under the fixed load condition.

1. Fixed load
  The system target speed of 2000 r/min is given as above, and a 3Nm load interference is applied to the system at 0.1 s. The comparisons of speed response and performance indicators are obtained in the operating system, as shown in Fig. 12 and Table 8, respectively. It can be seen from Fig. 12 and Table 8, when the load is added to the system, the undershoot of PID is the most obvious, and the volatility of FuzzyPID, PSO-FuzzyPID, GA-PID-FLC, DPNN-FuzzyPID, and DFPID-HSA is weak. Among them, the shortest Settling time of DFPID-HSA is about 0.001 s, and the smallest steady-state error is 4.5 r/min. It can be seen that DFPID-HSA is obviously better than other algorithms in terms of anti-interference ability.

**Table 8:** The comparison of performance indicators under the fixed load condition.

Controllers	Transient performance indicators			Steady-state performance indicators	
	Maximum undershoot (− Mp%) × 10^−5^	Peak time (s)	Settling time (s)	Steady-state error (r/min)	Steady-state error (%)
PID	2342.13	0.108	0.060	9.5	0.475
FuzzyPID	7.54	0.121	0.020	11.6	0.580
PSO-FuzzyPID	12.31	0.116	0.017	9.9	0.495
GA-PID-FLC	9.04	0.116	0.010	8.6	0.430
DPNN-FuzzyPID	9.19	0.115	0.009	7.5	0.375
DFPID-HSA	6.77	0.100	0.001	4.5	0.225
2. Variable load
  Next, there is a continuous sinusoidal signal load disturbance applied to the system, which is defined as $T_{m} = 20\sin t,\; 0 \le t \le 0.2s$. The comparison of the speed response and performance indexes under the operating system is shown in Fig. 13 and Table 9. From Fig. 13 and Table 9, it can be seen that the oscillation of PID is most obvious when the system is accompanied by sinusoidal signal load and causes severe steady-state errors. The fluctuation of FuzzyPID, PSO-FuzzyPID, GA-PID-FLC, and DPNN-FuzzyPID is weaker. Among them, DFPID-HSA has no obvious fluctuation phenomenon and still maintains the shortest settling time and the smallest steady-state error. It can be seen that DFPID-HSA has good robustness and anti-interference performance.

**Figure 13:**
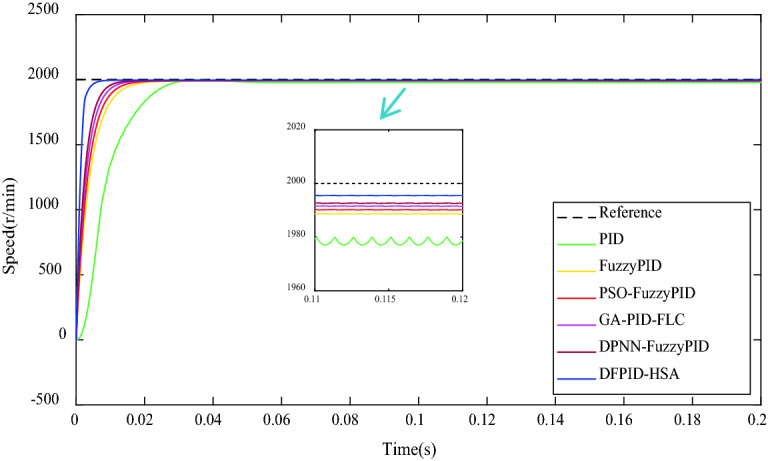
The comparison of speed response under the variable load condition.

**Table 9:** The comparison of performance indicators under the variable load condition.

Controllers	Transient performance indicators			Steady-state performance indicators	
	Maximum overshoot (Mp%)	Delay time (s) × 10^–3^	Settling time (s)	Steady-state error (r/min)	Steady-state error (%)
PID	0.95	7.20	0.085	21.0	1.050
FuzzyPID	–	2.65	0.050	11.6	0.580
PSO-FuzzyPID	–	2.37	0.046	10.2	0.510
GA-PID-FLC	–	1.83	0.040	8.6	0.430
DPNN-FuzzyPID	–	1.52	0.030	7.6	0.380
DFPID-HSA	–	0.60	0.015	4.3	0.215

### Speed changes condition

**Figure 14:**
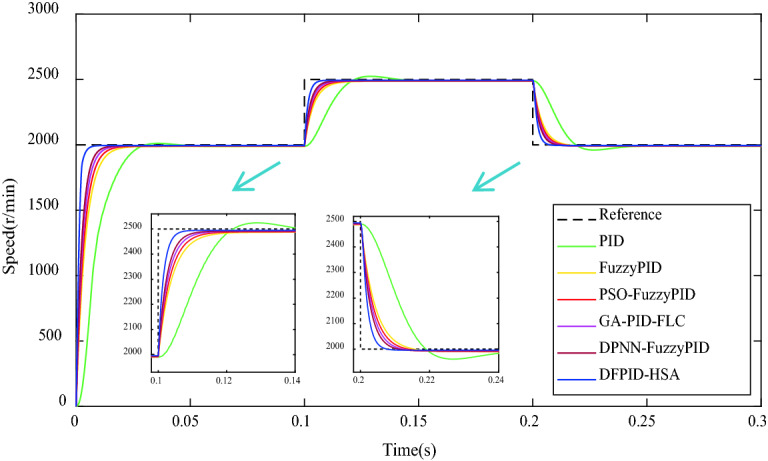
The comparison of speed response under the speed changes condition.

Speed changes condition is a common situation in the operation of BLDCM, so it is essential to verify the control performance of DFPID-HSA under this working condition. First, the initial target speed of the BLDCM system is given at 2000 r/min in the no-load state, and the speed is increased to 2500 r/min at 0.1 s, and then reduced to 2000 r/min again at 0.2 s. The corresponding comparison of speed response is shown in Fig. 14, and the comparison data of performance indicators are given in Table 10. It can be seen from Fig. 14 and Table 10, PID is still accompanied by an overshoot/undershoot phenomenon. FuzzyPID, PSO-FuzzyPID, GA-PID-FLC, DPNN-FuzzyPID, and DFPID-HSA have relatively good performance, but DFPID-HSA is optimal for the delay, settling, and steady-state error. Therefore, this proves the superiority of DFPID-HSA once again.

**Table 10:** The comparison of performance indicators under the speed changes condition.

Controllers (speed up/speed down)	Transient performance indicators			Steady-state performance indicators	
	Maximum overshoot/undershoot (Mp%) × 10^–2^	Delay time (s) × 10^–3^	Settling time (s)	Steady-state error (r/min)	Steady-state error (%)
PID	1.38/− 1.47	9.0/9.2	0.080/0.080	9.80/10.00	0.392/0.500
FuzzyPID	–/–	3.0/2.7	0.035/0.035	13.20/10.35	0.528/0.518
PSO-FuzzyPID	–/–	2.5/2.4	0.030/0.030	11.41/9.02	0.456/0.451
GA-PID-FLC	–/–	2.1/2.3	0.025/0.026	9.86/7.78	0.394/0.389
DPNN-FuzzyPID	–/–	1.8/1.9	0.020/0.022	8.59/6.78	0.344/0.339
DFPID-HSA	–/–	1.2/1.5	0.015/0.017	5.67/4.16	0.227/0.208

### Mechanical parameters variation conditions

**Figure 15:**
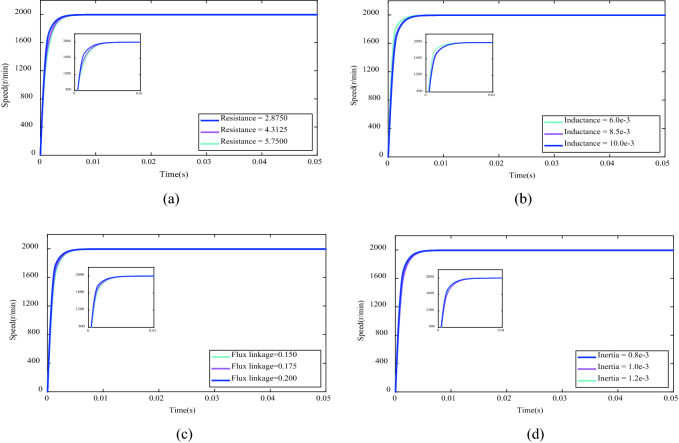
The comparison of speed response under the mechanical parameters variation conditions: (**a**) Resistance (**b**) Inductance (**c**) Flux linkage (**d**) Inertia.

In view of the optimization control problem of DFPID-HSA in this paper, it is essential to analyze the sensitivity of mechanical parameters variations of the BLDCM system. Here, the resistance, inductance, flux linkage, and inertia of the BLDCM system are adjusted for the corresponding increases or decreases, and the corresponding curves under the conditions of the relevant mechanical parameters variations are given in Fig. 15. As can be seen from the figures, even if the relevant mechanical parameters increase or decrease in amplitude, DFPID-HSA can still achieve speed tracking well, without overshoot/undershoot and oscillation. It only changes in the delay time and stability time, but this does not affect the final stability of the system. Hence, it can be certified that DFPID-HSA has excellent robustness.

## Experimental analysis

**Figure 16:**
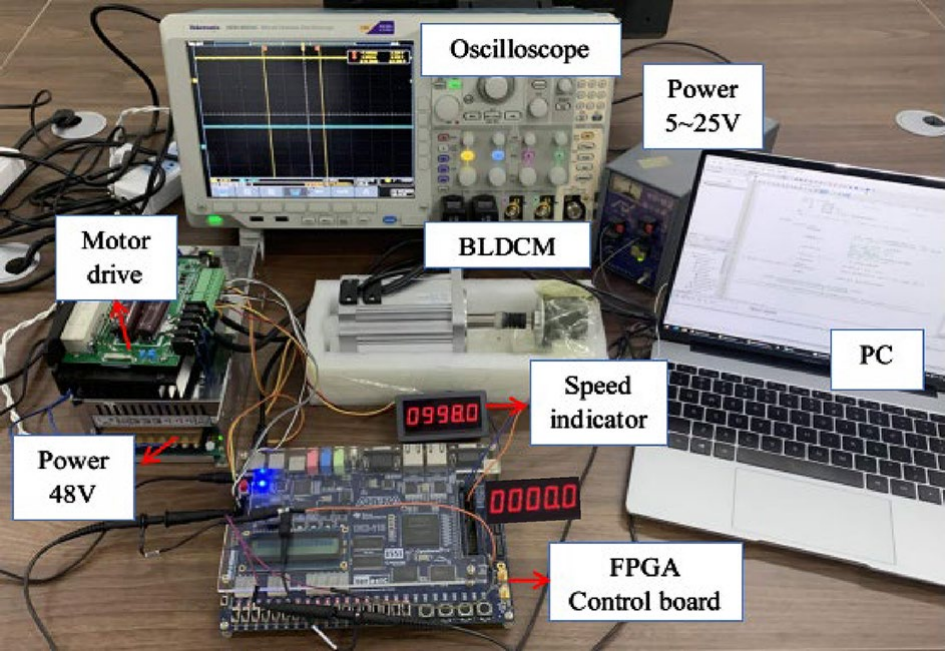
The experimental platform of the BLDCM control system.

To further verify the feasibility of DFPID-HSA, the experimental platform for the BLDCM control system is set up, as shown in Fig. 16. The BLDCM used in the testing platform is 80BL110S50-445TKA, and its driver adopts the IR2235 driver chip of the International Rectification Company. IR2235 is a high-voltage, high-speed MOSFET and IGBT drive circuit, with its current amplification and protection functions while suppressing noise at the output. In the experiment, an incremental encoder E6C2- CWZ5B with a resolution of 600 is used for speed detection. The control board model is DE2-115, and the FPGA chip model is EP4CE115F29C7. The oscilloscope is MDO4000C of TEKTRONIX Company. In the experiment, this paper uses the logical resources of FPGA to build a NIOS II soft-core processor, and the DFPID-HSA is programmed in the constructed NIOS II soft-core by C language to realize real-time control.

**Figure 17:**
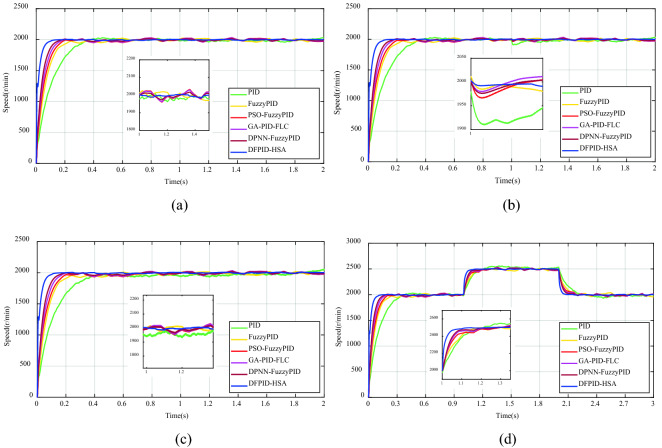
Experimental test results of DFPID-HSA: (**a**) No-load (**b**) Fixed Load (**c**) variable load (**d**) Speed changes.

Corresponding to the working conditions in the previous section, the algorithm is tested experimentally, and the experimental results are shown in Fig. 17. In the experiment, the target speed is still set at 2000 r/min, and the experiment time was mapped to 10 times. The external resistance is increased at 1 s to achieve a sudden load change, and the sudden change in speed is achieved at 1 s/2 s. The relevant parameters of each algorithm are appropriately scaled, and the optimization objective constraints in DFPI-HSA are adjusted to: $K_{P1} ,K_{I1} ,K_{D1} = [0,100]$, $k_{{p^{\prime}}} ,k_{{i^{\prime}}} ,k_{{d^{\prime}}} = [0,30]$. As can be seen from Fig. 17, the five algorithms can well realize speed tracking under no-load, fixed load, variable load, or speed changes conditions. However, compared with the simulation test, the algorithms in the experiment all have fluctuations phenomenon. It can be seen from (a) in Fig. 17 and Table 11, the overshoot phenomenon of PID is still apparent, and its fluctuation frequency is fast. The ranges of FuzzyPID, PSO-FuzzyPID, GA-PID-FLC, and DPNN-FuzzyPID are more significant, but the frequency of the fluctuations is slower. Compared with the above four algorithms, DFPI-HSA has the weakest fluctuation phenomenon, showing its good robustness. In the cases of the fixed load, variable load, and speed changes, the control effect of DFPID-HSA is relatively best. Overall, in the experiment, DFPID-HSA still maintains its superiority and can realize the excellent control of BLDCM.

**Table 11:** The comparison of performance indicators under different conditions.

Controllers/performance indicators	Oscillation range under no load (r/min)	Undershoot under fixed load (− r/min)	Oscillation range under variable load (r/min)	Settling time under speed changes (s)
**PID**				
Value	[1957.93, 2031.74]	90.04	[1930.95, 2046.15]	0.65
Percentage	3.69%	4.50%	5.76%	65%
**FuzzyPID**				
Value	[1949.95, 2025.35]	15.43	[1922.57, 2020.00]	0.30
Percentage	3.77%	0.77%	4.87%	30%
**PSO-FuzzyPID**				
Value	[1952.59, 2032.41]	30.68	[1932.77, 2032.05]	0.28
Percentage	3.99%	1.53%	4.96%	28%
**GA-PID-FLC**				
Value	[1956.17, 2029.20]	20.86	[1939.37, 2030.31]	0.25
Percentage	3.65%	1.04%	4.55%	25%
**DPNN-FuzzyPID**				
Value	[1969.79, 2016.94]	24.14	[1954.35, 2019.70]	0.20
Percentage	2.36%	1.21%	3.27%	20%
**DFPID-HSA**				
Value	[1986.79, 2005.78]	8.06	[1982.92, 2009.18]	0.16
Percentage	0.95%	0.40%	1.31%	16%

## Conclusion

In this paper, a novel PID controller using the dual fuzzy logic system with HSA optimization called DFPID-HSA is presented to enhance the speed control performance of BLDCM. The stability of the proposed controller is analyzed by the pole determination method, the Lyapunov determination method, and the Nyquist determination method. Then the system has been demonstrated to be closed-loop stable. To test and verify the superiority of DFPID-HSA, its performance is analyzed and compared with DPNN-FuzzyPID, GA-PID-FLC, PSO-FuzzyPID, FuzzyPID, and PID under the conditions of no-load, fixed load, variable load, and speed changes. The results show that DFPID-HSA is superior to other algorithms in the field of steady-state performance indicators, transient performance indicators, and integral performance indicators. In addition, the sensitivity analysis of DFPID-HSA is performed to evaluate its robustness under the condition of variable mechanical parameters. Finally, an experimental platform for the BLDCM drive system is built to further demonstrate the superiority and feasibility of DFPI-HSA in practical applications.

## Data Availability

Data analysis in the current study is available from the corresponding author on reasonable request.
